# Effects of the Addition of Hands-on Procedures to McKenzie Exercises on Pain, Functional Disability and Back Mobility in Patients with Low Back Pain: A Randomised Clinical Trial

**DOI:** 10.21315/mjms2023.30.3.11

**Published:** 2023-06-27

**Authors:** Lina Abdullah Ali Al-Banawi, Enas Fawzy Youssef, Alsayed Abdelhameed Shanb, Belal Elsayed Shanb

**Affiliations:** 1 Anak General Hospital, Eastern Health Cluster, Ministry of Health, Kingdom of Saudi Arabia; 2 Orthopedic Physical Therapy Department, Faculty of Physical Therapy, Cairo University, Egypt; 3 Department of Physical Therapy, College of Applied Medical Sciences, Imam Abdulrahman Bin Faisal University, Kingdom of Saudi Arabia; 4 Faculty of Medicine, Helwan University, Egypt

**Keywords:** low back pain, hands-on procedures, manual therapy, patients

## Abstract

**Background:**

Low back pain (LBP) is a common musculoskeletal disorder that affects people of all ages. This study investigates the effects of the addition of hands-on procedures to McKenzie exercises on patients with LBP and derangement syndrome.

**Methods:**

Forty-eight female patients were randomly assigned to either the experimental group or control group. All the patients in both groups underwent McKenzie exercises, transcutaneous electrical nerve stimulation (TENS) and education for 35 min/session–45 min/ session, with three sessions/week for 2 weeks. Hands-on procedures were added to the McKenzie extension exercises only for the patients in the experimental group. A visual analogue scale (VAS), the Oswestry disability index (ODI), back range of motion (BROM) and body diagrams were used to measure pain, functional disability, BROM and the centralisation of symptoms, respectively.

**Results:**

The mean values of VAS, ODI and BROM significantly improved after the interventions in both groups (*P* < 0.05), whereas the results of repeated measures ANOVA and Mann-Whitney U tests showed statistically non-significant differences between the two groups (*P* > 0.05).

**Conclusion:**

The addition of hands-on procedures to McKenzie exercises, TENS and education significantly alleviated back pain and functional disability and improved the back mobility and centralisation of symptoms in patients with LBP and derangement syndrome; however, these measures did not result in any significant additional benefits for such patients.

## Introduction

Low back pain (LBP) is a common musculoskeletal disorder that affects people of all ages—almost 80% of people experience it at one point in their lives (1). It usually forces the affected individual to seek the help of a health professional (2). LBP is characterised by pain, muscle tension or stiffness extending from below the costal margin to above the inferior gluteal folds, with or without referred leg pain. Due to the high prevalence of LBP and its complex affective interactions, its proper management requires multidisciplinary approaches comprising various medical specialties (3).

LBP is considered the leading cause of activity restriction and work absenteeism across the world (4). It is a major burden in terms of personal suffering and societal costs, including both productivity and healthcare costs (5, 6).

Derangement syndrome is a disturbance in the normal resting position of the joint surface that causes pain and restricts the mobility of the spine. Most patients who suffer from common mechanical spinal disorders fall under the category of derangement syndrome (7). This syndrome outlines seven types of derangement, each with specific characteristics and clinical symptoms that can be detected during the patients’ physical examination (8). For example, derangements one, three and five constantly occur without any postural deformities, whereas derangements four and six often present with a sciatic scoliosis (lateral shift) deformity (8).

Physical therapists play an important role in the assessment and treatment of LBP, such as pain reduction, function improvements and patient education, to avoid or reduce additional pain recurrences (9). Physical therapy (PT) includes both passive and active interventions (10). Passive interventions mainly use heat/ice packs, ultrasound and transcutaneous electrical nerve stimulation (TENS), whereas active interventions involve different forms of exercises, such as strengthening, stretching, aerobics, range of motion (ROM) and core or balance programme, such as Pilates (3). Active interventions are recommended more than passive interventions because of the former’s high efficacy in the treatment of LBP (11).

Manual therapy (MT) has been shown to have beneficial effects at all stages of LBP (12). The McKenzie method is a well-known MT used in the treatment of LBP. In this method, patients actively participate by performing exercises, correcting their posture and monitoring their symptoms (8). It has the potential to serve as the ideal classification system for patient treatment without documenting any adverse events (13, 14). In addition, the therapist uses hands-on procedures only if the patient fails to obtain satisfactory results (8).

Hands-on techniques are a combination of selected extension exercises and MT for certain areas of the spine (15). This manual force is used only for a small percentage of patients to eliminate their pain. Clinicians can apply manual pressure at the end range and even progress to spinal mobilisation and manipulation in the direction preference of the patient’s symptoms. Often, minimal force is required to achieve the desired effects of the centralisation of symptoms and pain elimination. Most patients can self-manage carrying out end-range exercises under the clinician’s guidance (14).

The McKenzie method is slightly more effective than back school interventions for disability treatment but not for reducing pain intensity (16). In addition, the McKenzie method is superior to placebo for pain treatment but not for disability treatment (17). The addition of McKenzie exercises to first-line care does not produce any further appreciable pain, disability alleviation or function improvement. No significant differences were reported in the outcome measures, such as back stiffness, ROM and pain, after the addition of posterior-to-anterior mobilisation to either the McKenzie method or other therapeutic interventions (18, 19). Although McKenzie exercises have positively contributed to the treatment of LBP patients, further studies are required to validate their effectiveness for patients with LBP and derangement syndrome, particularly those who participated in the current study (2). In addition, several authors have recommended conducting further research to evaluate the effects of handson McKenzie exercises on patients with LBP (8, 12, 19). Therefore, this study aims to investigate the effects of adding hands-on procedures to McKenzie exercises, TENS and education on the pain, functional disability, back mobility and centralisation of symptoms in patients with subacute or chronic LBP and derangement syndrome.

## Methods

All procedures were approved by the Ethics Research Committee of the Institutional Review Board of Imam Abdulrahman bin Faisal University, Saudi Arabia. This study was conducted in accordance with the Declaration of Helsinki at the Rehabilitation Clinic, Anak General Hospital, Ministry of Health, Dammam, Saudi Arabia, between January 2016 and January 2017. All patients signed a consent form. They were informed that the gathered data would be submitted for publication. Clinical trial registration: https://clinicaltrials.gov/ct2/show/NCT03066674

### Study Design

#### Randomised clinical trial

**Figure 1:**
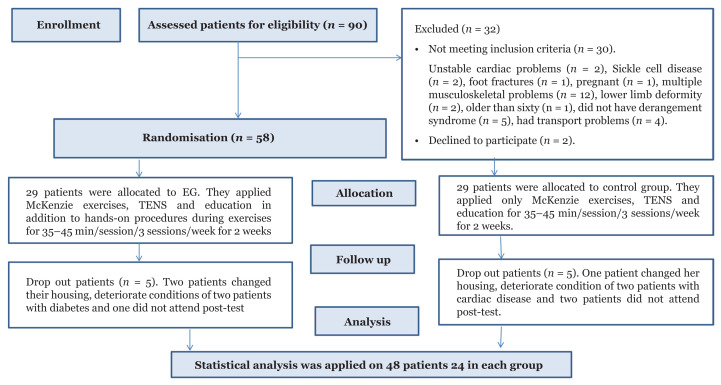
Flow chart of the patient’s recruitment

All patients were randomised to either the experimental group (EG) or the control group (CG). Pieces of paper were numbered and placed in a closed box. Each patient was instructed to pick one piece of paper from the box. Those who picked odd numbers were assigned to the EG, whereas those who picked even numbers were assigned to the CG (20) (Figure 1).

### Sample Size Calculation

The sample size was calculated using the https://www.stat.ubc.ca/~rollin/stats/ssize/n2.html website. The means and standard deviation (SD) of the pain measure ([μ1] = 1.53; [μ2] = 2.66; SD = (1.39)) were reported in a previous study (21). The statistical significance was set at 0.05, with a power of 0.80.

### Inclusion Criteria

Patients with subacute or chronic LBP with or without referred leg pain, who are 30 years old–60 years old and who have manifestations of derangement syndrome were included in this study (8, 22, 23). Derangement syndrome is characterised by pain due to severe strain, sustained flexion strain, difficulty assuming a comfortable sleeping position, history of recurrent LBP (particularly during flexion/ extension movements), frequent deviation of the trunk to one side, constant worse pain in the sitting position, possible presence of centralisation of symptoms and a lateral-shift deformity in the standing position (22, 23).

### Exclusion Criteria

Patients with serious fractures, tumours, ankylosing spondylitis, nerve root compromises, severe and unstable cardiopulmonary diseases, cervical or thoracic pain rather than lumbar pain, sickle cell disease, previous back surgery, severe osteoporosis, spinal instability and pregnancy were excluded from the study (23, 24).

### Assessment Procedure

Forty-eight female patients with LBP and derangement syndrome were recruited (Figure 1) after their diagnosis by an orthopaedic specialist at the Anak General Hospital Outpatient Clinic, Ministry of Health, Dammam, Saudi Arabia. All patients were asked to stop taking any medications for LBP management (25). Their body mass index (BMI) values were calculated. All patients received a standardised assessment for outcome measures conducted by a research assistant (assessor) blinded to the patients’ groups, and treatment procedures were conducted by the primary investigator, who was a certified McKenzie practitioner and had extensive experience in the treatment of orthopaedic cases.

Passive central posterior-to-anterior mobilisation (CPA) was applied to all participating patients by the primary investigator over the spinous process of each lumbar vertebra using a small amplitude (Grade I) from the prone position, starting with lumbar five and progressing to lumbar one (23). This mobilisation provoked pain and discomfort (12). If no pain was produced with Grade I, higher grades were applied (26). CPA mobilisation was repeated three times to determine the most painful level, which was considered the proper and accurate segmental level for the treatment (27, 28).

### Pain

Pain was measured using a visual analogue scale (VAS), which is a reliable measure for measuring the pain caused by musculoskeletal problems. Every patient placed a mark on the line representing their pain intensity level (29).

#### Functional disability

The percentage of disability in patients with LBP was estimated using the Arabic version of the Oswestry Disability Index (ODI) (29, 30). The patients marked the readings in the instrument on a scale of 0–5. The lowest score represents minimal disability and the highest score represents severe disability. The percentages of changes in the clinical indicators for VAS and ODI were estimated using the formula:


(A-B)/A×100%

where A is the pre-intervention value and B is the post-intervention value. A positive number indicates improvement, whereas a negative number indicates the opposite (31).

### Back range of motion

Back range of motion (BROM) was measured using a universal inclinometer (PA Deluxe, New York). Back forward flexion, extension and right- and left-side flexion were measured from the standing-erect position for every patient. The trunk’s right and left rotations were measured from the sitting position using a standard goniometer whose measurements were proven to be valid and reliable.

### Centralisation of symptoms

The locations of the symptoms were recorded on the patients’ body diagrams to determine the extent of the symptoms. This method has been shown to have excellent reliability (32).

### Therapeutic Procedure

#### Experimental group

Every patient underwent a therapeutic programme with the following components for 35 min/session–45 min/session, with three sessions/week for 2 weeks.

##### Transcutaneous electrical nerve stimulation (Chattanooga, UK)

Transcutaneous electrical nerve stimulation (TENS) was used as an analgaesic modality for back pain and applied from a prone lying position for 20 min at a frequency of 20 Hz–50 Hz and a wave duration of 50–100. Either two electrodes were placed on each side of the painful area, or two electrodes were placed on the painful area, and the other two electrodes were placed on the path of the radiating nerve (33). TENS is widely used for the treatment of musculoskeletal pain. The intensity of the stimulation was increased gradually until a tingling sensation and painless paraesthesia were felt by the patient (33).

##### Extension exercises

The following three exercises were performed by every patient in three sets, with 10 repetitions for 10 min–15 min (8, 27).

- Extension in standing: Every patient was asked to lean backwards as far as possible from a well-balanced standing position and to let their head tip back after arching backwards and returning to the neutral standing position.
- Extension in lying with a partial range: This was done from the prone lying position, with the hands under the shoulders to elevate the trunk, as a push-up exercise.
- Extension in lying with a full range: This was similar to the previous exercise in the partial range, but the arms were fully extended to achieve the maximum possible tolerated extension range (sustained movement for 1 s–2 s).

### Hands-on Procedures

**Figure 2:**
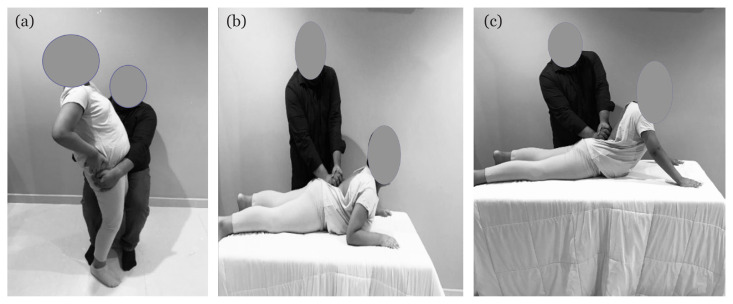
Addition of hands-on procedures to McKenzie exercises only for the McKenzie group A) Central posterior – anterior during extension in standing position B) Central posterior –anterior during partial extension range in prone lying C) Central posterior –anterior during full extension range in prone lying position

These are passive CPA procedures conducted over lumber spinous processes. They were applied only to the EG patients while performing the three afore mentioned extension exercises. The target was to improve the standardisation of the examiner’s exerted force according to Maitland grades (12). Grades I and II were used for the first three sessions to reduce pain and irritability, whereas Grades III and IV were used for the last three sessions to increase joint mobility for a total duration of 10 min–15 min (8, 28) (Figure 2).

#### Education

Every patient was educated regarding the treatment programme and was given instructions for posture correction for 5 min/ session–10 min/session. The education included basic information about back pain, its causes and guidance for exercises at home (emphasising good posture during prolonged sitting, standing, bending or twisting, lying down and resting positions) (16).

#### Home programme

Each patient received a brochure explaining the home exercise programme (8).

### Control group

Every CG patient applied all the EG therapeutic interventions except the addition of hands-on procedures while performing back extension exercises.

### Statistical Analysis

The collected data were analysed using SPSS statistical software (version 25.0). All data were tested for normality using a Shapiro-Wilk’s test. An independent *t*-test was used to compare the demographic data between the two groups. Repeated measures ANOVA was used to determine significant differences within each group and between groups for normally distributed variables, whereas McNemar and Mann-Whitney U tests were used to determine the significant differences within each group and between both groups for abnormally distributed variables. Statistical significance was set at (*P* < 0.05) with a confidence interval of 95%.

## Results

### Demographic Data

Of the 90 female patients with LBP and derangement syndrome, 48 (53%) completed this study. The number of patients who had subacute and chronic LBP was 21 (44%) and 27 (56%), respectively. As shown in Table 1, there were statistically non-significant differences in the demographic data between the EG and CG patients (*P* > 0.05).

### Back Pain, Disability Index and Centralisation of Symptom

The mean values of pain and the ODI (Table 2) show statistically significant reductions within each group after the interventions (*P* < 0.05). The pre- and post-values of the median (interquartile range) for the centralisation of symptoms were 2(0) and 1(1), respectively, for both groups. The number of patients who answered ‘Yes’ for centralisation changed from 2 to 17 and from 3 to 16 in the EG and CG, respectively. The results of McNemar’s test for the centralisation of symptoms show statistically significant differences after the interventions within each group (*P* < 0.001 for both). On comparing back pain and functional disability, the results of repeated measures ANOVA show statistically non-significant differences between the EG and CG patients (*F*-value = 0.002 and 1.939; *P* = 0.968 and 0.171), respectively. Furthermore, the results of Mann-Whitney U test show statistically non-significant differences in the centralisation of symptoms between EG and CG patients (*Z*-value = −579; *P* = 0.562).

### Back Range of Motion

Table 3 shows that there were statistically significant differences in the mean values of back flexion, extension and right and left lumbar rotations after the interventions (*P* < 0.05 for both). On comparison, the results of repeated measures ANOVA for back flexion and extension show that there were statistically non-significant differences between the EG and CG patients (*F*-value = 0.033 and 0.786; *P* = 0.856 and 0.380), respectively. In addition, there were non-statistically significant differences in the back-right and left rotations between the EG and CG patients (*F*-value = 0.620 and 0.713; *P* = 0.435 and 0.403), respectively.

Table 4 shows statistically significant increases only in right-side flexion of both EG and CG patients after the interventions (*P* < 0.05). On comparison of right-side flexion with left-side flexion, there were statistically non-significant differences between the EG and CG patients (*F*-value = 0.296 and 1.535; *P* = 0.589 and 0.222), respectively.

## Discussion

Despite technological advancements in new treatment modalities for LBP, it is still a common musculoskeletal disorder that affects people of all ages. The results of the current study show that the age and gender of patients may contribute to increases in the incidence rate of LBP. These results are supported by Chowdhury et al. (34), who found that the common age of patients with LBP ranged from 40 years old–59 years old and by Knauer et al. (35), who proved that the age of 14% of patients with chronic back pain was 45 years old–64 years. In addition, a higher rate of LBP was found in female patients, particularly after menopause, which could be the result of changes in their oestrogen hormone levels (36). Wang et al. (36) reported that after the age of 40, 60% of women with osteophytes and degenerative joint changes develop LBP. Unfortunately, the patient sample in the current study included only overweight female patients. The increases in body weight are supported by Chowdhury et al. (34), who found a strong positive association between body weight and the incidence rate of LBP.

The current study’s results show that the back pain and functional disability of the patients were alleviated in both groups, and their back mobility and the centralisation of symptoms improved significantly. The achieved percentages of pain reduction were 43.87% and 49.42% for the EG and CG patients, respectively. Both percentages are higher than the clinically important differences in pain reduction (30%) (31). These findings of pain reduction are in agreement with the findings of previous studies (18, 22, 27, 35, 37). Goodsell et al. (18) proved that CPA mobilisation resulted in 33% pain reduction in patients with LBP. In addition, Shah and Kage (27) compared CPA mobilisation with the McKenzie press-up. The authors obtained higher percentages of pain reduction (78.5% and 50%) than those obtained in the current study because they applied CPA mobilisation over the most painful segment in addition to other lumbar vertebral levels, whereas in the current study, CPA mobilisation was applied only over the most painful segment. Moreover, Chiradejnant et al. (37) found that two 1-min bouts of spinal mobilisation achieved 36% pain reduction in patients with LBP. The underlying mechanisms of pain reduction could be explained by the fact that the programme used in the current study was comprehensive. It included TENS, education and home exercises in addition to McKenzie exercises as an MT; these interventions have mechanical and neuro-physiological effects on pain reduction, minimise the dorsal horn activations from aching stimuli and lead to transient inhibitory effects on the alpha motor neurons (38). Spinal manipulation increases pain tolerance or its threshold (39). Manipulation can remove noxious mechanical or chemical stimuli from the paraspinal tissues and can also affect the reflex neural outputs to both the muscles and visceral organs (27). Repetitive mobilisation movements increase the spread of synovial fluid over the articular cartilage and disc, thereby creating less resistance to motion and smooth joint movements, which results in pain reduction (27). McKenzie extension exercises can also reduce pain by relocating the nucleus pulpous material (8, 40).

The ODI scores improved significantly in both groups after the interventions. The percentages of clinical improvement in ODI were 30% and 28% for the EG and CG patients, respectively. ODI score reduction usually represents a reduction in pain intensity parallel with increases in ROM and functional activities (alleviating functional disability) (27). This is compatible with the results of Clare et al. (41) and Garcia et al. (16), which positively support the effectiveness of the McKenzie programme in minimising back disability.

LBP is a serious health problem that imposes a huge burden on society. However, for its treatment, there are simple, low-cost and safe PT procedures, such as CPA spinal mobilisation and McKenzie exercises combined with other non-invasive interventions, such as TENS and education.

The BROM, including flexion, extension and rotation to both sides, improved significantly after the interventions in both groups. The increases in back extension ROM in both groups were in agreement with the findings of Shah and Kage (27), who found that the McKenzie programme and lumbar mobilisation mainly improved the extension lumbar ROM. They also found a positive relationship between increases in the back extension ROM and centralisation of symptoms (24). Our study’s results also show that there were significant increases only in right-side flexion in EG and CG patients after the interventions, and the back right and left rotations improved significantly in both groups. These results are consistent with the findings of Klein et al. (42), but there are differences in the mean values possibly because their sample of patients consisted of young males (18 years old); in the current study, the sample consisted of only adult females (> 40 years old). The achievement of these satisfactory results in terms of pain reduction and functional disability measures is expected to have direct positive effects on back mobility, BROM and the centralisation of symptoms.

Finally, the results of the current study show that the addition of hands-on procedures to McKenzie exercises did not lead to any significant additional benefits in pain, functional disability, back mobility and the centralisation of symptoms for the EG patients. These results are in accordance with the findings of Goodsell et al. (18) and Paatelma et al. (19). Goodsell et al. (18) reported that lumbar posterior-to-anterior mobilisation did not result in any significant measurable change in patients with LBP in their study. Furthermore, Paatelma et al. (19) reported that MT did not produce any significant differences compared with the McKenzie programme. In addition, Rasmussen et al. (43) did not find any significant additional differences in the comparison of manipulation with extension exercises in patients with non-specific LBP (43). The McKenzie programme always emphasises self-treatment with proper exercises for patients with non-specific LBP. For acute and chronic LBP, spinal manipulative therapy may not be more effective than passive interventions, sham manipulation or adjunct therapy (8). Therefore, it is likely that hands-on procedures may be used only if self-treatment fails to achieve the desired alleviations and improvements.

Contrary to our findings, Choi et al. (44) and Hidalgo et al. (12) support the benefits of using MT as mobilisation for patients with LBP, especially when MT is combined with flexion-distraction techniques (44) or an exercise training programme (12), or if it is used as an approach to treat LBP (31). Moreover, Marshall et al. (45) established that MT is better than traditional PT in pain and disability management. This may be because they applied only one exercise (static stretching of the hamstring muscles), whereas in the current study, a programme comprising three back exercises, TENS, home exercises and education was applied. However, to date, there is limited evidence of the effectiveness of adding MT to McKenzie exercises for the treatment of chronic LBP (12). It could be concluded that mobilisation and McKenzie exercises may result in the same end results and outcomes (43).

The percentage of improvement in the centralisation of pain was 71% and 67% for the EG and CG, respectively, which is in good agreement with the clinical observations of McKenzie and May (8) and Donelson et al. (46). In addition, the findings of Long et al. (22) revealed the occurrence of rapid changes in the pain location of patients with derangement syndrome. This is consistent with the concept that centralisation is associated with a good prognosis (7). Contrary to the results of the current study, Werneke et al. (32) found that the centralisation of symptoms is more common in adult patients with acute back pain than in older patients with chronic back problems; however, the participating patients in the current study had subacute and chronic LBP (44% and 56%), respectively.

## Conclusion

The addition of hands-on procedures to McKenzie exercises, TENS and education significantly alleviated back pain and functional disability and improved back mobility and the centralisation of symptoms in patients with LBP and derangement syndrome. However, it did not result in any significant extra benefits for such patients.

### Limitations

As the current study had a small sample size and included only female patients with LBP, its findings are not generalisable. In addition, the short duration of the interventions (only 2 weeks) resulted in the inability to evaluate the long-term effects of the interventions.

### Recommendations

Further studies are needed to investigate the short- and long-term effects of the addition of hands-on procedures to McKenzie exercises on both genders to generalise the findings of the current study and to analyse the associated psychosocial factors in patients with LBP and derangement syndrome.

## Figures and Tables

**Table 1:** Demographic data of the recruited patients (*N* = 48)

Variables		EG mean (SD)	CG mean (SD)	*P-*value
Age (years old)		48.46 (8.12)	47.67 (2.91)	0.655†^ª^
Weight (kg)		69.35 (6.63)	68.64 (6.93)	0.719†^ª^
Height (cm)		161 (0.61)	159 (0.07)	0.548†^ª^
BMI (kg/m^2^)		27.14 (1.43)	26.95 (1.57)	0.668†^ª^
Pain (VAS)		7.5 (1.84)	7.75 (1.15)	0.576†^ª^
Disability (ODI)		33.54 (13.29)	38.33 (8.52)	0.144†^ª^
Back pain *N* (%)	Sub-acute	8 (33%)	13 (54%)	0.150†^b^
	Chronic	16 (67%)	11 (46%)	

Notes:

a Independent *t*-test;

b Mann-Whitney test;

BMI = body mass index; CG = control group; EG = experimental group; *N* (%) = numbers (percentage); ODI = Oswestry disability index; SD = standard deviation; VAS = visual analog scale;

† = indicates statistically non-significant difference (*P*-value > 0.05)

**Table 2:** Back pain and disability index pre- and post-interventions in both groups

Variables	Pain (VAS)				Disability index (ODI)			
	
	EG		CG		EG		CG	
			
	Pre	Post	Pre	Post	Pre	Post	Pre	Post
Mean	7.5	4.21	7.75	3.92	33.54	23.58	38.33	27.42
SD	1.84	3.11	1.15	2.41	13.29	12.52	8.52	10.61
CI 95%	2.17 (4.41)		2.65 (5.02)		7.47 (12.44)		7.09 (14.74)	
*F*-value	37.158		44.732		68.687		34.846	
*P*-value a	* < 0.001		* < 0.001		* < 0.001		* < 0.001	

Notes:

a Repeated-measures ANOVA test was used to detect significant differences within each group;

CI = Confidence interval at 95%; ODI = Oswestry disability index; VAS = visual analog scale;

* Indicates statistically significant difference (*P*-value < 0.05)

**Table 3:** Lumbar range of motion pre- and post-interventions in both groups

Variables	EG		CG		EG		CG	
			
	Pre	Post	Pre	Post	Pre	Post	Pre	Post

ROM	Flexion of back				Extension of back			
Mean	40.88	70.62	45.21	64.96	8.58	16	8.96	17.08
SD	9.70	18.88	11.32	15.09	2.08	2.79	2.68	5.09
95% CI	−35.28 (−24.22)		−25.12 (−14.38)		−8.51 (−6.32)		−9.97 (−6.28)	
*F*-value	123.732		57.810		197.381		83.079	
*P*-value	*< 0.001		*< 0.001		*< 0.001		*< 0.001	

**ROM**	**Right lumbar rotation**				**Left lumbar rotation**			

Mean	42.88	49.79	45.67	51.0	47.25	54.21	49.79	59.21
SD	13.94	6.34	7.84	11.87	19.11	18.28	11.01	15.96
95% CI	−12.31 (−1.52)		−9.36 (−1.30)		−12.38 (−1.54)		−13.02 (−5.81)	
*F*-value	7.027		7.486		7058		29.243	
*P*-value^a^	*0.014		* 0.012		*0.014		* < 0.001	

Notes:

a Repeated measures ANOVA test was used to detect significant differences within each group;

* Indicates statistically significant difference (*P*-value < 0.05);

CG = control group; CI = confidence interval at 95%; EG = experimental group; SD = standard deviation

**Table 4:** Right and left side flexion pre- and post-interventions in both groups

Variables	Right side flexion of back				Left side flexion of back			
	
	EG		CG		EG		CG	
			
	Pre	Post	Pre	Post	Pre	Post	Pre	Post
Mean	14.79	17.12	13.63	17.33	15.29	15.12	15.38	17.17
SD	2.93	3.69	2.39	4.69	1.88	3.08	3.81	4.81
95% CI	−3.57 (−1.09)		−4.55 (−1.97)		−1.02 (1.35)		−3.81 (0.23)	
*F*-value	12.230		19.416		0.085		3.356	
*P*-value a	* 0.001		*< 0.001		†0.774		†0.080	

Notes:

a Repeated-measures ANOVA test was used to detect significant differences within each group;

CG = control group; CI = confidence interval at 95%; EG = experimental group; SD = standard deviation;

* Indicates statistically significant difference (*P*-value < 0.05);

† Indicates statistically non-significant difference (*P*-value > 0.05)
